# Dental Composition Modified with Aryloxyphosphazene Containing Carboxyl Groups

**DOI:** 10.3390/polym12051176

**Published:** 2020-05-20

**Authors:** Evgeniy M. Chistyakov, Natalya Kolpinskaya, Vera Posokhova, Vladimir Chuev

**Affiliations:** 1 D.Mendeleev University of Chemical Technology of Russia, Miusskaya sq. 9, 125047 Moscow, Russia; 2 Trade House VladMiVa, 308015 Belgorod, Russia; 3 Belgorod National Research University, 308015 Belgorod, Russia

**Keywords:** phosphazene, restorative dentistry, adhesion, composite material, modification

## Abstract

A modifier consisting of the mixture of cyclotriphosphazenes containing 4-allyl-2-methoxyphenoxy and β-carboxyethenylphenoxy moieties was developed for administration with acrylate dental restorative compositions. The synthesized compounds were characterized by ^1^H and ^13^C NMR spectroscopy and MALDI-TOF mass spectrometry. The optimal conditions to combine the modifier with the starting dental mixture consisting of bis-GMA and TGM-3 were revealed by differential scanning calorimetry (DSC) method. Properties of the cured modified compositions were evaluated for the compliance with requirements of ISO 4049:2019. It was found that these compositions possess the increased adhesion to dental tissues and cure depth and the decreased water sorption and water solubility. The values of elastic modules, destructive compressive stress and microhardness were also increasing along with the increased content of the modifier in the composition.

## 1. Introduction

The improvement of wellbeing is currently remaining one among the most important problems. First of all, it concerns the human health. Medicine [1], the pharmaceuticals industry [2,3], genetic engineering [4,5] and dentistry [6] are all hot topics among modern research trends. It should be noted that the restorative dentistry needs new materials possessing improved performance properties [7,8]. Most works are aimed at enhancing the mechanical properties and durability of dental polymer materials [9,10], their antibacterial properties [11,12,13,14,15] and their biocompatibility [12,16,17,18], as well as decreasing the shrinkage of composite [12,19,20]. There are several approaches to achieve these goals.

The development of new fillers is the most common way to improve the properties of dental composites. Thus, an application of zinc oxide nanoparticles was reported [16] to enhance the mechanical properties and antibacterial activity of the composite without reducing its biocompatibility. Another example to improve the mechanical characteristics is the utilization of hydroxyapatite nanorods [21] or hybrid nano- and microparticles of hydroxyapatite [18] as a filler. The combination of these particles with microspheres of dicalcium phosphate anhydrate [22] promotes an ability of the composite to mineralize and suppress caries. The improved mechanical properties and mineralization ability can be also observed upon an administration of modified zeolites [23]. Amorphous calcium phosphate nanoparticles added to the binder [24] ensure the possibility of multiple releasing of calcium and phosphate ions, which significantly increases the mineralizing ability. Titanium dioxide is a filler used commonly [11,12,25]. The nanoparticles enhance the antibacterial properties of the dental fillings without compromising mechanical properties, while its combination with calcium aluminate even improves these properties [25].

Another way to improve the properties of dental materials is through a modification of the binder. For instance, bisphenol A-glycidyl methacrylate was esterified with various aliphatic acid chlorides [19]. The resulting esters demonstrated lower viscosity and shrinkage upon their polymerization. The modification of bisphenol A-glycidyl methacrylate with a new photocurable monomer “Phene” facilitates a reduced polymerization shrinkage [20].

A current problem in restorative dentistry is the low adhesion of dental material on tooth tissues [26]. It is also possible to increase the adhesion via modifications of the binder [27]. Therefore, the present work is aimed at the design of a modified acrylate dental composition possessing improved performance properties. It was assumed that the presence of carboxyl groups in the modifier may enhance adhesion of the dental material to the tooth tissues, due to interactions of these groups with the dentin, while a large number of the double bonds in the modifier would ensure the formation of a dense spatial network with an acrylate binder, facilitating improvements in the physicochemical and physicomechanical properties of the resulting composite.

Within the framework of this work, the following tasks were set in order to achieve the desired result: synthesize a multifunctional modifier bearing carboxyl groups; estimate the optimal ratio of components and the appropriate conditions for obtaining a dental mixture; evaluate characteristics of the modified dental compositions. To this end, we decided to synthesize aryloxycyclotriphosphazene-containing carboxyl groups and double bonds capable of copolymerization with acrylates and to evaluate this compound as the modifier for dental compositions.

## 2. Materials and Methods

*Materials*. Hexachlorocyclophosphazene (HCP) (Fushimi Pharmaceutical Co., Ltd., Marugame, Kagawa Prefecture, Japan) was purified by the recrystallization from *n*-hexane with the subsequent sublimation. The starting stomatologic blend (BM) (Trade House VladMiVa, Belgorod, Russia) consisting of Bis-GMA (70%) and triethylene glycol dimethacrylate (30%) was used without any purification. Glass filler was also received from Trade House VladMiVa. Other reagents were purchased from Sigma-Aldrich (St. Louis, MO, USA). Eugenol, chloroform, pyridine, piperidine, 4-hydroxybenzaldehyde, camphorquinone, ethyl-4-dimethylaminobenzoate, K_2_CO_3_, CaCl_2_, NaOH and malonic and hydrochloric acids were used without further purification. Dioxane and THF were dried over CaH_2_ and distilled.

*Methods*. ^31^P NMR spectra were recorded on a Bruker Avance 300 spectrometer (Bruker, Billerica, MA, USA). ^1^H and ^13^C NMR spectra were recorded on an Agilent/Varian Inova 400 spectrometer (Agilent Technologies, Santa Clara, CA, USA). Mass spectrometry data were acquired using a Bruker Auto Flex II mass spectrometer (Bruker, Billerica, MA, USA). DSC analysis was performed using a NETZSCH STA 449F1 instrument (10 K/min, Ar) (Netzsch, Selb, Germany). The compositions were cured using a Rainbow Curing Light dental photopolymerizer based on the LED technology (Foshan Fibor, Foshan, China). The compositions were irradiated for 40 s at the wavelength of 420–480 nm using the energy source of 1000–1200 mW cm^–2^. The flexural strength, water absorption, solubility and cure depth were measured according to the requirements ISO 4049:2019. Adhesive strength was estimated according to ISO 4049:2009. The modulus of elasticity was determined according to ISO 4049:1988. The compression strength was estimated according to ISO 604:2002. The flexural strength, cure depth, adhesive strength, the modulus of elasticity and the compression strength were measured using an LRX universal testing machine (Lloyds Instruments, Ametek, Berwyn, IL, USA). The Vickers hardness was measured 24 h after the light curing using a micro-indentation tester (Shimadzu Micro Hardness Testers HMV-2 (Shimadzu, Kyoto, Japan) for a 100-gf load applied for 40 s. The measurements were performed at depths of 1, 2, 3, 4 and 5 mm from the upper surface.

*Statistical analysis*. Average values of the performance characteristics of various samples were compared using two-way ANOVA followed by the Tukey’s special analysis for multiple comparisons.

*Synthesis of product I*. Eugenol (0.66 mL, 0.0043 mol) was dissolved in THF (50 mL) under stirring in a round bottomed flask (100 mL) equipped with a stirrer, finely-ground K_2_CO_3_ (1.19 g, 0.0086 mol) and HCP (0.5 g, 0.00144 mol) were then added. The reaction was refluxed for 5 h, the precipitate was filtered off, and the solvent was distilled off. Chloroform was added to the resulting residue, and the solution was washed with 0.1-M NaOH and then with distilled water. The solution was dried over CaCl_2_, CHCl_3_ was distilled off and product I was dried in vacuo. The yield was 0.87 g (82.7%). ^1^H and ^13^C NMR spectra (recorded in CDCl_3_) are given in the Supplementary Materials.

*Synthesis of product II*. Eugenol (0.66 mL, 0.0043 mol) was dissolved in dioxane (50 mL) under stirring in a round bottomed flask (100 mL) equipped with a stirrer, finely-ground K_2_CO_3_ (1.19 g, 0.0086 mol) and HCP (0.5 g, 0.00144 mol) were then added.

The reaction was carried out as 101 °C for 5 h, 4-hydroxybenzaldehyde (0.53 g, 0.0043 mol) was then added to the reaction mixture and the reaction was refluxed for additional 8 h. The precipitate was separated by centrifugation and the supernatant solution was precipitated by pouring into water. The product was dissolved in chloroform and washed with 0.1-M NaOH and then with distilled water. Chloroform was distilled off and the product was dried in vacuo until a constant weight. The yield was 2.68 g (90.5%). ^1^H and ^13^C NMR spectra (recorded in CDCl_3_) are given in the Supplementary Materials.

*Synthesis of product III*. Malonic acid (3.8 g, 0.0365 mol) and product II (4.5 g, 0.0046 mol) were placed into a round bottomed flask (50 mL) equipped with reflux condenser and stirrer, pyridine (10 mL) was added and piperidine (one drop) was then added. The reaction was refluxed until the end of carbon dioxide emission. The resulting product was precipitated in hydrochloric acid (1 M, 200 mL). The aqueous layer was decanted, washed with water and dried in vacuo until a constant weight. The yield was 4.45 g (87%). ^1^H and ^13^C NMR spectra (recorded in DMSO-*d*_6_) are given in the Supplementary Materials.

*Preparation of compositions modified by product III*. Product III and BM were placed into a round bottomed flask equipped with a stirrer at the ratios given in Table 1. The flask was filled with Ar and the mixture was heated at 60 °C under stirring until product III was completely dissolved. The modified binder (50 g) was obtained for each ratio of components.

The compositions were prepared by mixing the glass filler (77 wt %) and binder (33 wt %) in a vacuum mixer–homogenizer. A mixture (0.37 wt %) of camphorquinone and ethyl 4-aminobenzoate taken at the molar ratio of 1:2 was introduced as the photoinitiator system. All the components were loaded subsequently, after which the mixture was stirred for at room temperature 2 h. The numbers of the obtained compositions correspond to the numbers of binder samples. The obtained compositions were placed into molds and cured. Various molds were used in order to obtain the samples of different shapes and sizes, which was necessary for their evaluations according to the corresponding standards.

## 3. Results and Discussion

### 3.1. Synthesis and Characterization of the Modifier of Dental Composition

**Figure 1:**
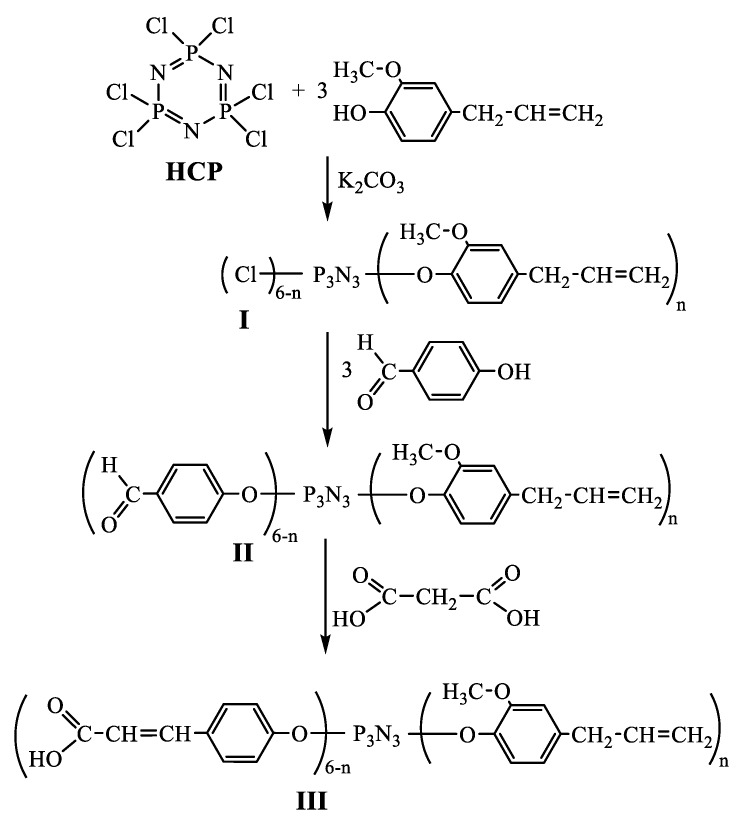
Synthetic scheme of cure site monomer preparation for the dental composition (I, II, III—products, the synthesis of which is indicated in the section “Materials and Methods”).

To ensure a good adhesion to dental tissue, the composition has to contain groups capable of interacting with hydroxyapatite. As such groups, carboxyl ones can be employed. Their introduction can be performed via a copolymerization of the composition monomers with carboxylic acids containing carbon–carbon double bonds. However, it is undesirable in the case of photocurable dental fillers to use acids bearing only one group capable of the polymerization. First of all, because the polymerization proceeds via a chain mechanism and is characterized by a significant content of the residual monomer. The second reason is the decreased density of crosslinked polymer, which negatively affects mechanical properties of the composition. Therefore, the employment of polyfunctional monomers, which maintain a good chemical interaction with the both dental tissue and material of the dental composition, is preferable. Hexakis-(β-carboxyethenylphenoxy)cyclotriphosphazene—containing the six carboxyl and six double C=C bonds—is of interest as such a modifying monomer [28]. However, it was found that this compound is insoluble in the starting acrylate dental composition. It was suggested that this is caused by the high content of carboxyl groups in its molecule forming strong hydrogen bonds. Consequently, it was proposed to replace a part of β-carboxyethenylphenoxy substituents with 4-allyl-2-methoxyphenoxy groups containing the multiple bonds capable of polymerization. As a result, a cure site monomer (product III) soluble in the dental composition was synthesized. Figure 1 shows the scheme of its synthesis.

The ratio of groups in the modifier was set as 1:1, since the excessive content of β-carboxyethenylphenoxy moieties decrease the solubility in acrylic monomers, while a decreased content of these groups reduces the adhesion of material to the dental tissue.

**Figure 2:**
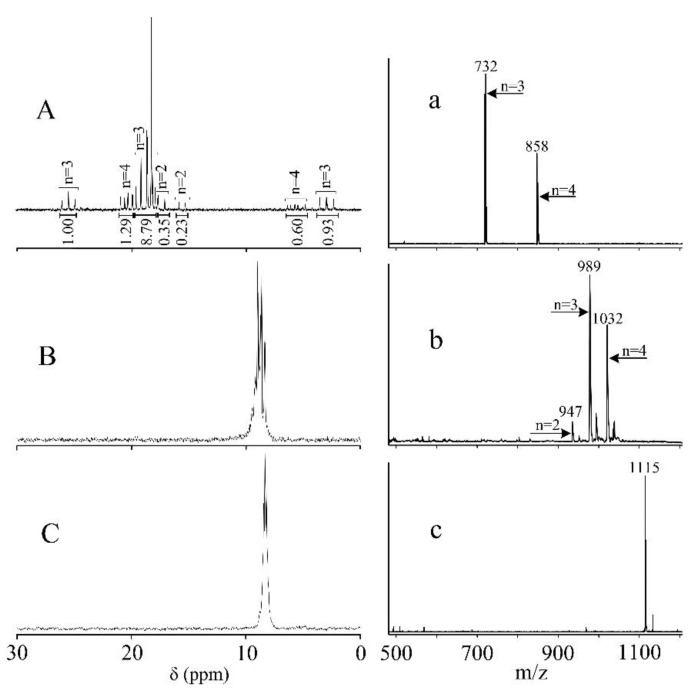
^31^P NMR (**A**, **B** and **C**) and MALDI-TOF (**a**, **b** and **c**) spectra of products I, II and III, respectively.

Product III was synthesized in three steps. Half of the chlorine atoms in hexachlorocyclotriphosphazene (HCP) were initially replaced with 4-allyl-2-methoxyphenoxy moieties via the reaction with eugenol. As one can see from the ^31^P NMR spectrum (Figure 2A), resulting product I was a mixture of homologs possessing different degrees of the chlorine substitution in HCP, where this degree of chlorine substitution n varies in the range from 2 to 4.

Amounts of the each homolog were estimated on the basis of integral intensities. They were 5% (compound where n = 2), 81% (n = 3) and 14% (n = 3). At the same time, the MALDI-TOF spectrum of product I (Figure 2a) does not contain any peak corresponding to a molecular ion of the homolog with n = 2. However, there is a peak corresponding to the compound containing two 4-allyl-2-methoxyphenoxy groups in the mass spectrum of product II (Figure 2b). This fact is probably due to the low content and higher reactivity of di-(4-allyl-2-methoxyphenoxy)-tetrachlorocyclotriphosphazene than the homologs possessing a higher degree of chlorine substitution in HCP. Consequently, side reactions such as hydrolysis and acidolysis caused by the matrix components were possible during the MALDI-TOF analysis. Such reactions lead to various rearrangements and destruction of the phosphazene ring [29,30].

At the second step, all the chlorine atoms in product I were replaced with 4-formylphenoxy groups via the reaction with 4-hydroxybenzaldehyde. ^31^P NMR spectrum of obtained product II contains only the one multiplet (Figure 2B), which indicates the completeness of the substitution reaction. The presence of multiplet instead of a singlet in the region of 8–10 ppm can be explained by a long-range order interaction between the phosphorus atoms in the phosphazene cycle. It is known that the formyl group exhibits the −M effect, while the methoxy one causes the +M effect. Consequently, the substituents bearing these groups cause different effects on the phosphorus atoms adjacent to them. Since product II contain the compounds possessing different values n, cis- and trans-isomers, geminal and non-heminal derivatives; all of them will form the different combinations of substituents at the phosphazene cycles. Each of these combinations exhibits its specific value of the chemical shift, which is slightly different from the chemical shift of other homologs. However, molecular ions of three derivatives can be clearly identified in the MALDI-TOF spectrum of product II: (946+H^+^) for n = 2, (988+H^+^) for n = 3 and (1031+H^+^) for n = 4 (see Figure 2b). This also confirms the completed replacement of all the chlorine atoms in product I.

**Figure 3:**
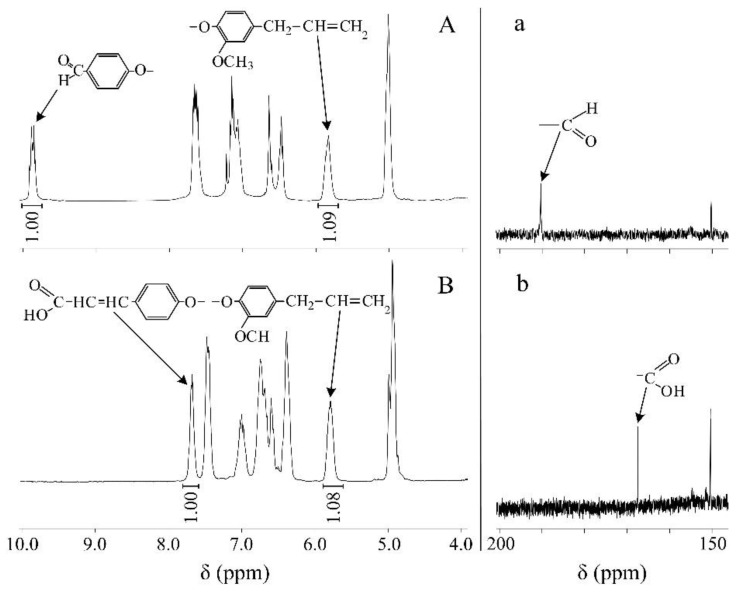
^1^H NMR (**A** and **B**) and ^13^C NMR (**a** and **b**) of compounds II and III, respectively.

The third step was a reaction to convert the formyl groups into β-carboxyethenyl ones. The reaction was carried out similarly to the known procedure [28]. The ^31^P NMR spectrum of obtained product III (Figure 2C) contains a multiplet (8–9 ppm), but it is narrower than that of product II. This is caused by a smaller effect of the β-carboxyethenylphenoxy group on the adjacent phosphorus atom in comparison with the 4-formylphenoxy group. Consequently, the interaction of long-range orders between the phosphorus atoms of phosphazene ring is less pronounced. The completeness of conversion of the formyl groups into the β-carboxyethenyl group was confirmed by MALDI-TOF spectrometry. The spectrum (Figure 2c) contains one signal corresponding to the molecular ion of product III (m/z = 1114+H^+^). There was only the one signal due to the absolutely identical molecular masses of the β-carboxyethenylphenoxy- and 4-allyl-2-methoxyphenoxy- moieties. The formation of β-carboxyethenyl groups was confirmed by ^1^H and ^13^C NMR spectroscopy. Comparison of the ^1^H NMR spectra of products II (Figure 2A) and III (Figure 2B) allows one to notice that the proton signal of the formyl group present in product II is absent in the spectrum of product III. At this end, the signal from one of the protons at the double bond of β-carboxyethenyl group can also be clearly distinguished in the spectrum of product III. Since the ratio of the integral intensities between this proton and the proton of the allyl group in product III (Figure 3B) is equal to the such ratio between the formyl group and the proton of the allyl group in product II (Figure 3A), it can be concluded that the transformation of the formyl groups into the β-carboxyethenyl group proceeded quantitatively. At this end, the total content of 4-allyl-2-methoxyphenoxy groups in product III was higher by about 8% than that of the β-carboxyethenylphenoxy groups. This was caused by a higher content of homologs where n = 4 than that of homologs where n = 2 in product III (Figure 2A). The presence of carboxyl groups in product III could not be confirmed by the ^1^H NMR spectrum due to the proton-deuterium exchange between DMSO-*d*_6_ and protons of carboxyl groups. Their formation was confirmed by ^13^C NMR spectroscopy. As one can see from Figure 3, the signal from the carbon atom of the formyl group (Figure 3A) of product II is disappeared in the spectrum of product III, while a carbon atom of the carboxyl group appears instead of it.

Therefore, we can conclude that the prepared modifier fully corresponds to the formula of product III shown in Figure 1, and the ratio between the 4-allyl-2-methoxyphenoxy and β-carboxyethenylphenoxy moieties in this product is approximately the same.

### 3.2. Properties of the Dental Compositions Modified with Product III

**Figure 4:**
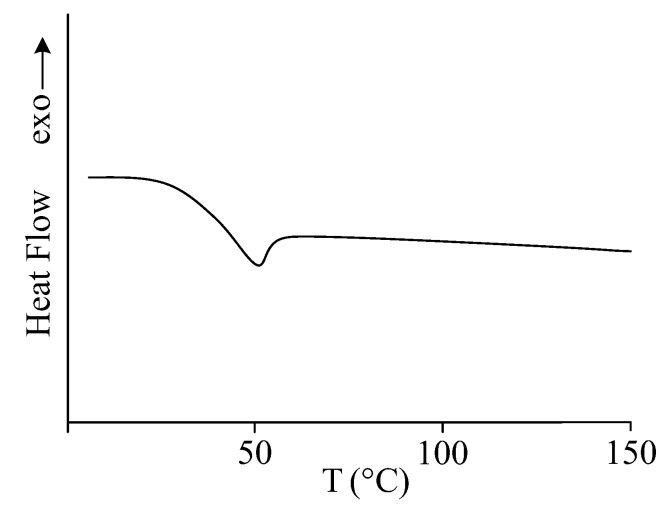
DSC curve for product III.

To evaluate the effect of product III on properties of the compositions, it was necessary to prepare solutions at different concentrations of the modifier in BM. This preparation was complicated by the solid state of product III at room temperature. The dissolution takes a very long time (from 3 to 5 days depending on the concentration) even in the case of fine grounded product. DSC analysis performed for product III allowed us to resolve this problem. It was found that the product is a completely amorphous substance possessing the glass transition temperature of 35–55 °C (Figure 4). Therefore, to reduce the time of dissolution of the product, it is optimal to carry out the process at a temperature above 55 °C, at which the substance is in the viscous-flow state.

At 60 °C, which is an allowed temperature in the case of BM components, the dissolution time was 4–10 h depending on the concentration.

To obtain the compositions, mixtures containing the following amounts of the modifier in BM were prepared: 1, 2.5, 5, 7.5 wt % and 10 wt %. However, it was impossible to prepare the mixtures containing more than 10 wt % of the modifier due to the limited solubility of product III in BM. The resulting binders were mixed with the filler, which are dressed glass microspheres.

First of all, the cured compositions were evaluated for the compliance with the requirements of ISO 4049:2019. The designed material is supposed to be applied as a material classified as Type 1, Class 2 and Group 1. According to this classification, the restorative dental material must meet the requirements given in Table 2. The same Table shows the test results acquired for the compositions based on BM and modified compositions.

As one can see from Table 2, the modifier content causes virtually no effect on the flexural strength. At the same time, the flexural strength values of the samples have completely met the requirements of ISO 4049:2019.

It is noteworthy that the water sorption and water solubility of the composition were significantly decreased as compared to those of composition based on pure BM (sample 0) even if only 1 wt % of product III was added in BM (sample 1). Further increases in the modifier content in the composition cause almost no effect on these parameters. This is probably due to the high content of double bonds in the modifier, which is already enough at its content of 1 wt % in the binder to form a dense network structure during the polymerization process. A high degree of crosslinking hinders the diffusion of substances from the composition and also prevents the penetration of water into it despite the high content of hydrophilic carboxyl groups present in the modifier. The obtained dental composites possess a lower water solubility than that of commercial light-cured glass ionomers, such as Vitremer and Fuji II LC [31]. In comparison with light-curing bulk-fill composites of the X-tra Fil, Tetric N-Ceram Bulk Fill and Filtek Z250 brands, the composites reported herein exhibit lower both water solubility and water sorption [32].

**Figure 5:**
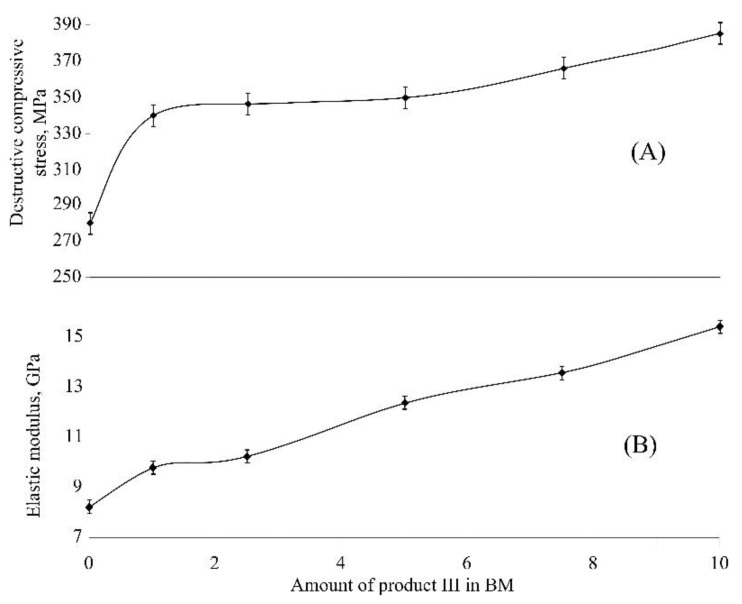
Dependences of (**A**) destructive compressive stress and (**B**) elastic modulus on the content of product III in the compositions.

The formation of a dense polymer network and its influence on properties of the compositions can be confirmed by a character of the dependence of destructive compressive stress vs. amount of product III in BM (Figure 5A). In the case of BM doped with 1 wt % of product III, the destructive compressive stress of composition was dramatically increased from 280 to 340 MPa as compared to the reference composition (sample 0). A further increase in the content of product III in the composition causes only a slight increase in this parameter.

One can see that the cure depth increases along with increasing the content of product III in the composition (Table 2), which consequently allows us to conclude that the lifetime of free radicals is elongated. This is probably caused by a steric factor due to the bulky molecules of product III, which hinder the bimolecular termination of growing chains during the polymerization.

As was initially expected, the presence of carboxyl groups in the composition improves its adhesion to the dental tissue. One can see from Table 2 that a higher content of the modifier in the composition provides the higher adhesive strength. In the case of BM doped with 10 wt % of product III, the adhesion of the composition increases by more than 6 times as compared with the reference composition based on pure BM.

Elastic modules of the compositions are linearly increased along with increasing the content of product III (Figure 5B). The value of elastic module for the composition containing 10 wt % of the modifier in the binder is higher by 88% than that value for pure BM.

**Figure 6:**
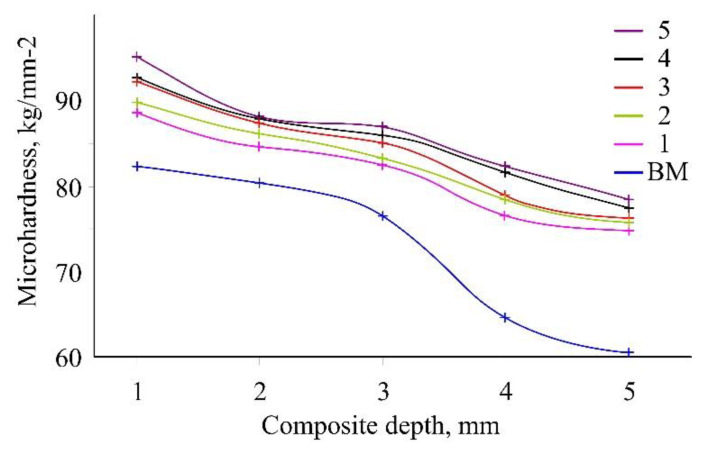
Microhardness at various depths for the cured compositions containing different amounts of the modifier. Numbers of lines correspond to the sample numbers.

Vickers microhardness of the cured compositions was investigated at depths of the material varying from 1 to 5 mm (Figure 6). As one can see from this Figure, the microhardness values decrease for all the samples along with going in the depth of material. This is fairly understandable for the case of radical polymerization processes initiated on the surface of material. Upon the irradiation, the initiation proceeds more intensively in the layer closest to the light source, and the polymerization is predominantly going in the upper layers of the material to form a denser polymer network and, thereby, to increase the hardness. This assumption is in a good agreement with other data [33] acquired previously for several non-modified commercial light-cured dental materials.

However, values of the microhardness in the entire depth range increase upon increasing the modifier content in the composition. This can also be explained by an increased density of polymer network due to the copolymerization of BM monomers with product III containing 6 double C=C bonds per molecule.

It is worth to note that a modification does not always provide positive results. Attempts to improve particular properties lead sometimes to degradation of the others [34]. However, the key result of this work is the synthesis of such modifier that has significantly improved almost all the physicochemical and physicomechanical characteristics necessary for a high-quality restoration material. An application of this modifier in combination with new dental achievements [35] may allow one to design later even more advanced materials.

## 4. Conclusions

In conclusion, this work demonstrated that a specially designed product consisting of a mixture of cyclotriphosphazenes bearing 4-allyl-2-methoxyphenoxy and β-carboxyethenylphenoxy moieties is the efficient modifier for dental acrylate compositions. The modified compositions completely met all requirements ISO 4049:2019 for dental restorative materials belonging to Type 1, Class 2 and Group 1.

Moreover, their performance properties were significantly improved with increased modifier content. As was expected, the content of carboxyl groups increased the adhesion of the dental material to the tooth tissues. In the case of binder doped with 10 wt % of the modifier, the adhesive strength increased by six times over that of the non-modified binder.

Although the requirements ISO 4049:2019 do not specify any standards for the elastic modules, destructive compressive stress and microhardness of dental compositions, these values were estimated in the present work. The values of these parameters are very important, since the restorative materials of the said type are subjected to regular mechanical loads during chewing of food. The developed modifier contributes to the increase in all three mentioned parameters of the cured dental compositions.

Taking into account everything presented hereinabove, it can be concluded that the developed dental composition can be practically applied as a high-quality, highly adhesive restorative material.

## Figures and Tables

**Table 1:** Ratio of components in the mixture used for obtaining the modified binder.

Binder Sample Number	Product III Content, wt %	Weight of Product III, g	Weight of BM, g
1	1	0.5	49.5
2	2.5	1.25	48.75
3	5	2.5	47.5
4	7.5	3.75	46.25
5	10	5	45

**Table 2:** Requirements ISO 4049:2019 for the restorative dental materials and results of the tests for the cured dental compositions. Adhesion was measured according to ISO 4049:2009.

Sample No.	Flexural Strength, MPa	Water Sorption, μg/mm^3^	Water Solubility, μg/mm^3^	Cure Depth, mm	Adhesion, MPa
0*	96.4 ± 2.8	17.2 ± 0.3	4.8 ± 0.1	2.49 ± 0.01	2.5 ± 0.1
1	97.2 ± 2.8	10.5 ± 0.2	2.4 ± 0.1	2.54 ± 0.01	3.6 ± 0.1
2	98.4 ± 3.0	10.6 ± 0.2	2.4 ± 0.1	2.58 ± 0.01	4.6 ± 0.1
3	104.5 ± 2.9	11.0 ± 0.2	2.5 ± 0.1	2.59 ± 0.01	9.2 ± 0.1
4	97.8 ± 2.8	11.0 ± 0.2	2.5 ± 0.1	2.75 ± 0.01	10.8 ± 0.1
5	106.5 ± 2.6	11.0 ± 0.2	2.5 ± 0.1	2.88 ± 0.01	15.4 ± 0.1
Requirements ISO 4049:2019	Not less 80	No more 40	No more 7.5	Not less 1.5	–

* Reference composition based on BM without added modifier.

## Supplementary Materials

The following are available online at https://www.mdpi.com/2073-4360/12/5/1176/s1, Figure S1: ^1^H NMR spectra of synthesized products. The numbers indicate the hydrogen atoms in the compounds and the signals corresponding to them, Figure S2: ^13^C NMR spectra of synthesized products. The numbers indicate the carbon atoms in the compounds and the signals corresponding to them, Table S1: Microhardness at various depths for the cured compositions containing different amounts of the modifier. Numbers of lines correspond to the sample numbers.
